# A cross-sectional analysis of the effects of residential greenness on blood pressure in 10-year old children: results from the GINIplus and LISAplus studies

**DOI:** 10.1186/1471-2458-14-477

**Published:** 2014-05-20

**Authors:** Iana Markevych, Elisabeth Thiering, Elaine Fuertes, Dorothea Sugiri, Dietrich Berdel, Sibylle Koletzko, Andrea von Berg, Carl-Peter Bauer, Joachim Heinrich

**Affiliations:** 1 Institute of Epidemiology I, Helmholtz Zentrum München, German Research Centre for Environmental Health, Ingolstädter Landstr, 1, 85764 Neuherberg, Germany; 2 Division of Metabolic and Nutritional Medicine, Dr. von Hauner Children’s Hospital, Ludwig-Maximilians-University of Munich, Lindwurmstraße 4, 80337 Munich, Germany; 3 School of Population and Public Health, The University of British Columbia, 2206 East Mall, V6T 1Z3 Vancouver, Canada; 4 IUF – Leibniz Research Institute for Environmental Medicine, University of Düsseldorf, Auf'm Hennekamp 50, 40225 Düsseldorf, Germany; 5 Research Institute, Department of Paediatrics, Marien-Hospital Wesel, Pastor-Janßen-Straße 8, 46483 Wesel, Germany; 6 Division of Paediatric Gastroenterology and Hepatology, Dr. von Hauner Children’s Hospital, Ludwig-Maximilians-University of Munich, Lindwurmstraße 4, 80337 Munich, Germany; 7 Department of Pediatrics, Technical University of Munich, Boltzmannstraße 15, 85748 Munich, Germany

**Keywords:** Greenness, NDVI, Blood pressure, Children, Green spaces

## Abstract

**Background:**

According to Ulrich’s psychoevolutionary theory, contact with green environments mitigates stress by activating the parasympathetic system, (specifically, by decreasing blood pressure (BP)). Experimental studies have confirmed this biological effect. However, greenness effects on BP have not yet been explored using an observational study design. We assessed whether surrounding residential greenness is associated with BP in 10 year-old German children.

**Methods:**

Systolic and diastolic BPs were assessed in 10 year-old children residing in the Munich and Wesel study areas of the German GINIplus and LISAplus birth cohorts. Complete exposure, outcome and covariate data were available for 2,078 children. Residential surrounding greenness was defined as the mean of Normalized Difference Vegetation Index (NDVI) values, derived from Landsat 5 TM satellite images, in circular 500-m buffers around current home addresses of participants. Generalized additive models assessed pooled and area-specific associations between BP and residential greenness categorized into area-specific tertiles.

**Results:**

In the pooled adjusted model, the systolic BP of children living at residences with low and moderate greenness was 0.90 ± 0.50 mmHg (p-value = 0.073) and 1.23 ± 0.50 mmHg (p-value = 0.014) higher, respectively, than the systolic BP of children living in areas of high greenness. Similarly, the diastolic BP of children living in areas with low and moderate greenness was 0.80 ± 0.38 mmHg (p-value = 0.033) and 0.96 ± 0.38 mmHg (p-value = 0.011) higher, respectively, than children living in areas with high greenness. These associations were not influenced by environmental stressors (temperature, air pollution, noise annoyance, altitude and urbanisation level). When stratified by study area, associations were significant among children residing in the urbanised Munich area but null for those in the rural Wesel area.

**Conclusions:**

Lower residential greenness was positively associated with higher BP in 10 year-old children living in an urbanised area. Further studies varying in participants’ age, geographical area and urbanisation level are required.

## Background

Over half of the world’s population lives in urban areas [1] and are thus often exposed to higher levels of environmental stressors (i.e. crowding, air and heat pollution, noise) than individuals living in rural areas. Contact with nature is believed to mitigate stress, as has been demonstrated in recent epidemiological studies [2-5]. Good access to structured green spaces as well as a high overall level of vegetation at residences (i.e. greenness) have been shown to alleviate stress [2-5].

The mechanisms by which green environments lead to stress restoration have been described, in particular, by Ulrich’s psychoevolutionary theory [6]. This theory argues that because human species have evolved over a long period in natural environments, they are physiologically and possibly psychologically better adapted to natural settings than artificial urban settings. Exposure to nature, according to Ulrich, induces an affective response by numerous physiological systems that leads to a reduction of sympathetic outflow measures, such as heart rate, cortisol level and blood pressure (BP) [6].

In line with Ulrich’s theory, exposure to nature scenes in laboratory settings and walking in green environments have been shown to lower BP, although marginally, in several experimental studies conducted in adults [7-12]. However, to date, no single study has investigated the effects of green spaces or greenness on BP using an observational study design. We aimed to fill this research gap by exploring whether greenness around the current home residence is associated with BP in 10 year-old German children. We believe children may be a particularly useful study population as they do not often take medications that can influence their BP, unlike adults.

## Methods

### Study population

The current analyses are based on data from two ongoing German birth cohorts: the “German Infant Study on the Influence of Nutrition Intervention plus Environmental and Genetic Influences on Allergy Development” (GINIplus) and the “Influence of Life-Style Factors on the Development of the Immune System and Allergies in East and West Germany plus the Influence of Traffic Emissions and Genetics” (LISAplus). Both cohorts have similar study designs and recruited only healthy full-term neonates with a normal birth weight. GINIplus participants were recruited in the cities of Munich and Wesel between 1995 and 1998 (N = 5,991). This cohort consists of two study groups: an observational study group and a study group that participated in an intervention trial with hypoallergenic formulae [13,14]. LISAplus participants were recruited in the cities of Munich, Leipzig, Wesel and Bad Honnef between 1997 and 1999 (N = 3,097) [15,16]. The GINIplus and LISAplus studies have been approved by their local ethics committees (Bavarian General Medical Council, University of Leipzig, Medical Council of North-Rhine-Westphalia) and informed consent was obtained from all parents of participants.

The current analyses are restricted to participants who resided in the Munich and Wesel study areas both at birth and at the 10 year follow-up. Furthermore, home address, BP and covariate data were required for inclusion. The final study population is comprised of 2,078 participants (N = 1,256 (52.1% male) from the Munich study area and N = 822 (50.7% male) from the Wesel study area).

### Greenness

Greenness was assessed using the Normalized Difference Vegetation Index (NDVI), derived from Landsat 5 Thematic Mapper (TM) satellite images (http://earthexplorer.usgs.gov/). NDVI is a common indicator of green vegetation which was developed to analyse surface reflectance measurements. The NDVI is based on two vegetation-informative bands (near-infrared (NIR) and visible red (RED)) and is calculated using the formula: NDVI = (NIR – RED)/(NIR + RED). NDVI values range from −1 to +1, with +1 indicating a high density of green leaves, −1 representing water features and values close to zero referring to barren areas of rock, sand or snow [17].

**Figure 1:**
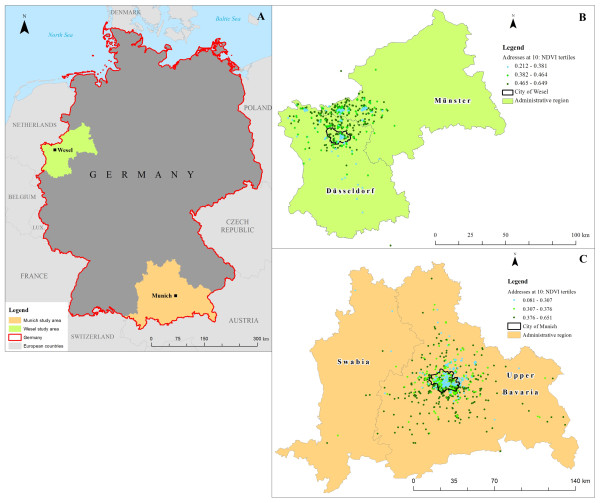
Location of the Munich and Wesel study areas on the map of Germany (A) and spatial distribution of the NDVI tertiles among participants residing in the Wesel (B) and Munich (C) study areas.

For both the Munich and Wesel study areas, we chose cloud-free images taken during summer months in 2003 for the NDVI calculation. For the Munich area, which includes two administrative regions of Bavaria (Upper Bavaria and Swabia; Figure 1), we used three images (two from the 14^th^ of July and one from the 24^th^ of August) as data covering the entire study area were not available for a single day. These three images were merged to obtain complete coverage of the Munich study area. For the Wesel study area, which includes two administrative regions of North-Rhein Westphalia (Münster and Düsseldorf; Figure 1), we used one image from the 10^th^ of July. Based on these images, NDVI values were calculated at a resolution of 30 m by 30 m. Negative values of NDVI were excluded before further calculation. Residential surrounding greenness was defined as the mean of NDVI values in a circular 500 m buffer around each participant’s home address. A 500 m buffer should represent a distance reachable within 10 minutes of walking [18] and is assumed to be a proxy for a child’s neighbourhood, as children are not as mobile as adults [19]. Previous studies on greenness and child and adult health, some of which were conducted by our group, have used this same buffer size [18,20-22].

Data management and calculations of NDVI were conducted using the ArcGIS 10.0 Geographical Information System (GIS) (ESRI, Redlands, CA) and Geospatial Modelling Environment (GME) (Spatial Ecology LLC) softwares.

### Blood pressure

Systolic and diastolic arterial BPs in children were measured during a physical examination at the 10-year follow up of the cohorts (2005 to 2009). The standardized protocol followed was the same as used in the population-based German Health Interview and Examination Survey for Children and Adolescents (KiGGS 2003–2006) [23]. Briefly, the first BP measurement was taken on the right arm when the child was in a sitting position and had rested for five minutes. A second measurement was taken after sitting for a further two minutes. The cuff size was selected according to the length and circumference of the upper arm of each child; the width was at least 2/3 the length and the pressure bladder covered at least half of the circumference of the upper arm. For the current analyses, an average of the two valid BP measurements was used, regardless of their difference. All measurements were performed by one physician in Munich and a second one in Wesel between 7:00 a.m. and 8:30 p.m. using an automatic BP monitor (Omron M5 Professional). A more detailed methodological description of the BP measurements for the two cohorts has been previously published [24,25].

### Covariates

Data on the following covariates were extracted from parent-completed questionnaires: study (GINIplus observational group/GINIplus interventional group/LISAplus), sex (male/female), parental education (both parents with < 10 years of school (low)/at least one parent with 10 years of school (medium)/at least one parent with > 10 years of school (high), according to the German education system) and parental hypertension (neither of the parents had hypertension, one of the parents had hypertension). The exact child’s age (in years) and the season (winter/spring/summer/autumn) at the time of the BP measurements were also available. The height and weight of each child at 10 years were also obtained when the BP measurements were conducted and used to calculate body mass index (BMI, kg/m^2^).

### Statistical analyses

Associations between residential surrounding greenness, estimated by NDVI, with systolic and diastolic BP were assessed using generalized additive models (GAMs; R package mgcv). GAMs allow the estimation of non-linear relationships between continuous predictor variables and a dependent variable via smooth functions [26]. Relationships between NDVI and BP were not linear and the pattern differed across the two study areas (Additional file 1: Figure S1). Therefore, we categorized NDVI values into tertiles in order to obtain numerical effect estimates and capture the pattern of the relationship, while maintaining a sufficient number of observations in each category, as has been previously done [27]. Associations between all continuous covariates and outcome variables were tested for linearity by incorporating the covariates as smooth terms. In the case of a non-linear relationship between a covariate and an outcome variable, the covariate was analyzed as a smooth term. Associations were investigated in the pooled study population using area-specific NDVI tertiles and adjustments for study centre and all covariates described in the above section. Area-specific models were also examined and adjusted for the same covariates, but study centre was excluded. All analyses were conducted using the statistical software R, version 3.0.2 [28].

### Further analyses: impact of environmental stressors

To explore whether associations between surrounding residential greenness and BP are influenced and/or mediated by environmental stressors, we conducted the following additional analyses.

#### Temperature

Outdoor temperature is known to influence both BP [29] and vegetation. Due to the high correlation between season and temperature, we were unable to include both covariates into a single model. Therefore, we excluded season from the models and instead adjusted for mean daily temperature on the day of each measurement.

#### Altitude

Ten year-old children residing at a high altitude have been reported to have higher BPs compared to those living at a low altitude [30]. As the terrain of the two current study areas differs significantly (the Wesel area is flat while the altitudes of the Munich residences vary from 369 m to 945 m), we additionally adjusted our models for altitude of residence but excluded study centre from pooled models to avoid collinearity. Altitude values were calculated at a 90 m spatial resolution using the Shuttle Radar Topography Mission (SRTM) dataset (http://dds.cr.usgs.gov/srtm/version1/Eurasia/).

#### Level of urbanisation

To test whether the effect of residential greenness on BP reflects a possible urbanisation effect, we adjusted our models for centre-specific population density tertiles derived in 5,000 m buffers around the residences, as has been done in a previous study by our group [31]. Data for these calculations were obtained from the WiGeoGIS population density dataset at a spatial resolution of 125 m for the year 2008.

#### Air pollution

In a previous analysis by our group, air pollution was not found to be associated with BP [32]. Nevertheless, we explored the potential role of air pollution on the association between greenness and BP by additionally adjusting the models for individual-level estimates of particulate matter (PM; including PM_2.5_ and PM_10_) and nitrogen dioxide (NO_2_) modelled to the home address of each child at 10 years of age. These air pollution estimates were derived using land use regression models developed separately for the Munich and Wesel areas as part of the European Study of Cohorts for Air Pollution Effects (http://www.escapeproject.eu/) [33-36].

#### Road traffic noise annoyance

In an experimental study, vegetation was reported to attenuate perceived traffic noise by affecting an individual’s emotional processing [37]. Therefore, we adjusted the models for road traffic noise annoyance, as reported by the parents of participants in the 10 year follow-up questionnaire. The original eleven category scale (from 0 to 10) was recoded into three levels of road traffic noise annoyance: low (0 and 1, no annoyance at all), medium (categories 2 to 5) and high (6 to 10, strong to unbearable annoyance) [38].

### Sensitivity analyses

Since BP is known to have circadian variation, we additionally adjusted the models for time of BP measurement (7:00 to 11:00/11:01 to 14:00/14:00 to 20:30) for the participants for whom this information was available (N = 1,091 (52.7 %)). Furthermore, we excluded participants who were living at their current address for less than one year (remaining N = 1,988, as 90 participants had moved within the previous 12 months). Additionally, we conducted stratified analyses between non-movers vs. movers between 6 and 10 years and between birth and 10 years. Moreover, in order to explore whether physical activity influences the association between greenness and BP, we additionally adjusted the models for parent-reported child physical activity. Finally, to assess the stability of the NDVI estimates, we chose alternative cloud-free days in 2006 to calculate a second set of NDVI values which were used to replicate all analyses (two images from 20^th^ of June and one from the 13^th^ for the Munich area and one image from the 18^th^ of July for the Wesel area; N = 2,036, because 42 addresses in the Munich area were covered by clouds).

## Results

Study participant characteristics and their exposure levels are provided for the pooled population and by study area in Table 1. The average systolic and diastolic BP in the pooled population was 111.3 ± 10.0 mmHg and 64.2 ± 7.5 mmHg, respectively. Children residing in the Wesel study area had a statistically significantly higher mean systolic BP compared to those living in the Munich area (113.2 ± 10.2 mmHg and 110.0 ± 9.6 mmHg, respectively). The same was true for diastolic BP (66.0 ± 7.0 mmHg and 62.9 ± 7.5 mmHg, respectively). The Wesel study participants differed from the Munich participants in numerous characteristics: they were slightly older, had higher BMI, and there were more participants from low SES families, all of which may result in higher BPs in Wesel. Average residential greenness, as measured by NDVI, was significantly higher in the more rural Wesel area than in the urbanised Munich area (0.430 ± 0.083 and 0.347 ± 0.089, respectively).

**Table 1:** Characteristics of the study participants

	**N (%) or Mean ± SD**^**a **^**or Median (IQR**^**b**^**)**		
	**Pooled (N = 2,078)**	**Munich (N = 1,256)**	**Wesel (N = 822)**
Systolic blood pressure (mmHg)^§^*	111.3 ± 10.0	110.0 ± 9.6	113.2 ± 10.2
Diastolic blood pressure (mmHg)^§^*	64.2 ± 7.5	62.9 ± 7.5	66.0 ± 7.0
Study*			
GINIplus observational group	773 (37.2)	370 (29.5)	403 (49.0)
GINIplus interventional group	754 (36.3)	448 (35.6)	306 (37.2)
LISAplus	551 (26.5)	438 (34.9)	113 (13.8)
Sex			
Male	1,071 (51.5)	654 (52.1)	417 (50.7)
Female	1,007 (48.5)	602 (47.9)	405 (49.3)
Age (years)^§^*	10.2 ± 0.2	10.2 ± 0.2	10.3 ± 0.2
BMI (kg/m^2^)^§^*	17.4 ± 2.5	17.0 ± 2.3	18.0 ± 2.7
Season of blood pressure measurements			
Winter	418 (20.1)	272 (21.7)	146 (17.7)
Spring	495 (23.8)	302 (24.0)	193 (23.5)
Summer	615 (29.6)	356 (28.3)	259 (31.5)
Autumn	550 (26.5)	326 (26.0)	224 (27.3)
Parental education^c^*			
Low	135 (6.5)	55 (4.4)	80 (9.7)
Medium	538 (25.9)	224 (17.8)	314 (38.2)
High	1,405 (67.6)	977 (77.8)	428 (52.1)
Parental hypertension^d^			
Yes	316 (15.2)	202 (16.1)	114 (13.9)
No	1,762 (84.8)	1,054 (83.9)	708 (86.1)
Residential surrounding greenness (NDVI^e^)^§^* in a 500 m buffer around the residence			
Low	-	0.255 ± 0.045	0.340 ± 0.035
Moderate	-	0.341 ± 0.020	0.425 ± 0.024
High	-	0.445 ± 0.057	0.524 ± 0.041
Mean daily temperature on the day of blood pressure measurements (°C)^¶^*	12.0 (10.4)	10.9 (11.3)	13.0 (9.1)
Altitude of residence (m)^¶^*	498.0 (509.0)	532.0 (44.0)	26 (17)
Population density around residence (people/km^2^)^§f^*			
Low	-	304.2 ± 152.0	176.2 ± 47.8
Moderate	-	1,558.6 ± 702.5	352.0 ± 93.1
High	-	5,479.8 ± 1,747.7	883.8 ± 292.7
PM_2.5_ concentration at residence (μg/m^3^)^§^*	14.9 ± 2.2	13.3 ± 0.9	17.4 ± 0.7
PM_10_ concentration at residence (μg/m^3^)^§^*	22.2 ± 3.3	20.0 ± 2.3	25.4 ± 1.3
NO_2_ concentration at residence (μg/m^3^)^§^*	21.3 ± 4.8	19.8 ± 5.1	23.6 ± 3.0
Road traffic noise annoyance^g^			
Low	1,367 (65.8)	821 (65.4)	546 (66.4)
Medium	608 (29.2)	371 (29.5)	237 (28.8)
High	103 (5.0)	64 (5.1)	39 (4.8)

^a^. SD: Standard deviation.

^b^. IQR: Interquartile range.

^c^. Low: < 10 years of school; Medium: 10 years of school; High > 10 years of school, according to the German education system.

^d^. No: neither of the parents had hypertension; Yes: one or both parents had hypertension.

^e^. NDVI: Normalized Difference Vegetation Index; categorized into tertiles.

^f^. Population density derived in 5,000 m buffers around residences and categorized into tertiles.

^g^. Low: categories 0 and 1 (no annoyance at all when the window is open); Medium:

categories 2 to 5; High: categories 6 to 10 (strong or unbearable annoyance).

^§^. Mean ± SD.

^¶^. Median (IQR).

*indicates statistically significant difference at the 5 % level between the Munich and Wesel areas.

Associations between residential greenness with systolic and diastolic BP are presented in Table 2. Crude and adjusted associations were similar. In the adjusted model, children residing in places with low levels of greenness had systolic BPs that were on average 0.90 ± 0.50 mmHg higher (p-value = 0.073) than the BPs of children living in areas with high levels of greenness, although this result was not statistically significant. Children with moderate levels of greenness had systolic BPs that were on average 1.23 ± 0.50 mmHg higher (p-value = 0.014) than the BPs of children living in areas with high levels of greenness. In this adjusted model, study centre, BMI, season of BP measurement and parental hypertension were significant terms. Similarly, children with low and moderate levels of greenness had diastolic BPs that were on average 0.80 ± 0.38 mmHg (p-value = 0.033) 0.96 ± 0.38 mmHg (p-value = 0.011) higher than the BPs of children living in areas with high levels of greenness. In this adjusted model, study centre, BMI, sex and parental hypertension were significant terms. Effect estimates remained robust when models were individually adjusted for environmental stressors (Table 2).

**Table 2:** Associations between residential greenness, defined using Normalized Difference Vegetation Index (NDVI), and systolic and diastolic blood pressure

**Model**	**NDVI**^**a**^	**Systolic blood pressure**			**Diastolic blood pressure**		
		**ß**^**b**^	**SE**^**c**^	**p-value**	**ß**^**b**^	**SE**^**c**^	**p-value**
**Crude**^**d**^	*Low*	**1.07**	**0.53**	**0.043**	**0.80**	**0.39**	**0.041**
	*Moderate*	**1.32**	**0.53**	**0.012**	**1.01**	**0.39**	**0.010**
	*High*	ref.	-	-	ref.	-	-
**Adjusted**^**e**^	*Low*	0.90	0.50	0.073	**0.80**	**0.38**	**0.033**
	*Moderate*	**1.23**	**0.50**	**0.014**	**0.96**	**0.38**	**0.011**
	*High*	ref.	-	-	ref.	-	-
**Temperature-adjusted**^**f**^	*Low*	0.83	0.50	0.098	**0.83**	**0.38**	**0.027**
	*Moderate*	**1.12**	**0.50**	**0.025**	**1.00**	**0.38**	**0.008**
	*High*	ref.	-	-	ref.	-	-
**Altitude-adjusted**^**g**^	*Low*	0.79	0.50	0.115	0.71	0.38	0.061
	*Moderate*	**1.16**	**0.50**	**0.021**	**0.90**	**0.38**	**0.017**
	*High*	ref.	-	-	ref.	-	-
**Urbanisation-adjusted**^**h**^	*Low*	0.82	0.53	0.125	0.73	0.40	0.069
	*Moderate*	**1.11**	**0.52**	**0.032**	**0.87**	**0.39**	**0.024**
	*High*	ref.	-	-	ref.	-	-
**PM**_**2.5 **_**–adjusted**^**i**^	*Low*	0.73	0.52	0.161	**0.93**	**0.39**	**0.017**
	*Moderate*	**1.13**	**0.51**	**0.026**	**1.03**	**0.38**	**0.007**
	*High*	ref.	-	-	ref.	-	-
**PM**_**10 **_**–adjusted**^**j**^	*Low*	0.96	0.54	0.074	**1.15**	**0.40**	**0.004**
	*Moderate*	**1.26**	**0.51**	**0.014**	**1.14**	**0.38**	**0.003**
	*High*	ref.	-	-	ref.	-	-
**NO**_**2 **_**–adjusted**^**k**^	*Low*	0.85	0.54	0.118	**1.01**	**0.41**	**0.014**
	*Moderate*	**1.20**	**0.52**	**0.021**	**1.08**	**0.39**	**0.006**
	*High*	ref.	-	-	ref.	-	-
**Noise annoyance-adjusted**^**l**^	*Low*	0.90	0.50	0.074	**0.80**	**0.38**	**0.034**
	*Moderate*	**1.23**	**0.50**	**0.015**	**0.97**	**0.38**	**0.011**
	*High*	ref.	-	-	ref.	-	-

^a^. Greenness tertiles are area-specific. Munich: Low (NDVI < 0.307), Moderate (0.307 ≤ NDVI < 0.377), High (NDVI ≥ 0.377). Wesel: Low (NDVI < 0.382), Moderate (0.382 ≤ NDVI < 465), High (NDVI ≥ 0.465).

^b^. ß: Regression coefficient.

^c^. SE: Standard error.

^d^. Crude model is adjusted for study centre.

^e^. Adjusted model is adjusted for study centre, study, sex, age, BMI, season of BP measurements, parental education, and parental hypertension.

^f^. Temperature-adjusted model: Adjusted model and further adjustment for mean daily temperature on the day of BP measurements. The variable for the season of BP measurements has been removed.

^g^. Altitude-adjusted model: Adjusted model and further adjustment for altitude of residence. The variable for the study centre has been removed.

^h^. Urbanisation-adjusted: Adjusted model and further adjustment for study-specific tertiles of population density in a 5,000 m buffer around the residence.

^i^. PM_2.5_-adjusted model: Adjusted model and further adjustment for PM_2.5_ concentration at residence.

^j^. PM_10_-adjusted model: Adjusted model and further adjustment for PM_10_ concentration at residence.

^k^. NO_2_-adjusted model: Adjusted model and further adjustment for NO_2_ concentration at residence.

^l^. Noise annoyance-adjusted model: Adjusted model and further adjustment for level of noise annoyance.

Bold text indicates statistical significance at the 5 % level.

Study-specific associations between greenness with systolic and diastolic BP are presented in Table 3. In the adjusted model for the Munich area, children with low and medium levels of greenness had systolic BPs that were on average 1.02 ± 0.63 mmHg (p-value = 0.106) and 2.22 ± 0.63 mmHg (p-value < 0.001) higher, respectively, than the BPs of children living in areas with high levels of greenness. Similarly, children with low and medium levels of greenness had diastolic BPs that were on average 1.08 ± 0.50 mmHg (p-value = 0.030) and 1.46 ± 0.50 mmHg (p-value 0.004) higher, respectively, than the BPs of children living in areas with high levels of greenness. All estimates for the Wesel area were not significantly different from null.

**Table 3:** Associations between residential greenness, defined using Normalized Difference Vegetation Index (NDVI), and systolic and diastolic blood pressure in Munich and Wesel study areas

**Model**	**NDVI**^**a**^	**Munich (*n*** **= 1,256)**						**Wesel (*n*** **= 822)**					
		**Systolic blood pressure**			**Diastolic blood pressure**			**Systolic blood pressure**			**Diastolic blood pressure**		
		**ß**^**b**^	**SE**^**c**^	**p-value**	**ß**	**SE**	**p-value**	**ß**	**SE**	**p-value**	**ß**	**SE**	**p-value**
**Crude**	*Low*	1.08	0.66	0.099	**1.06**	**0.51**	**0.039**	1.05	0.88	0.229	0.40	0.60	0.509
	*Moderate*	**2.33**	**0.66**	**< 0.001**	**1.47**	**0.51**	**0.004**	-0.21	0.88	0.814	0.31	0.60	0.609
	*High*	ref.	-	-	ref.	-	-	ref.	-	-	ref.	-	-
**Adjusted**^**d**^	*Low*	1.02	0.63	0.106	**1.08**	**0.50**	**0.030**	0.63	0.80	0.435	0.47	0.57	0.411
	*Moderate*	**2.22**	**0.63**	**< 0.001**	**1.46**	**0.50**	**0.004**	-0.26	0.80	0.746	0.22	0.57	0.696
	*High*	ref.	-	-	ref.	-	-	ref.	-	-	ref.	-	-
**Temperature-adjusted**^**e**^	*Low*	0.96	0.63	0.128	**1.05**	**0.50**	**0.035**	0.60	0.80	0.453	0.56	0.57	0.324
	*Moderate*	**2.19**	**0.63**	**0.001**	**1.43**	**0.50**	**0.004**	-0.46	0.80	0.564	0.37	0.57	0.516
	*High*	ref.	-	-	ref.	-	-	ref.	-	-	ref.	-	-
**Altitude-adjusted**^f^	*Low*	0.68	0.66	0.304	0.97	0.52	0.063	0.62	0.81	0.443	0.45	0.57	0.433
	*Moderate*	**2.01**	**0.64**	**0.002**	**1.39**	**0.51**	**0.006**	-0.29	0.80	0.719	0.17	0.57	0.764
	*High*	ref.	-	-	ref.	-	-	ref.	-	-	ref.	-	-
**Urbanisation-adjusted**^**g**^	*Low*	0.95	0.66	0.149	**1.03**	**0.52**	**0.049**	0.56	0.87	0.517	0.40	0.62	0.521
	*Moderate*	**2.09**	**0.66**	**0.001**	**1.36**	**0.52**	**0.009**	-0.37	0.82	0.649	0.17	0.58	0.767
	*High*	ref.	-	-	ref.	-	-	ref.	-	-	ref.	-	-
**PM**_**2.5 **_**–adjusted**^**h**^	*Low*	0.96	0.63	0.130	**1.16**	**0.50**	**0.021**	0.37	0.95	0.693	0.35	0.67	0.601
	*Moderate*	**2.18**	**0.64**	**0.001**	**1.53**	**0.50**	**0.002**	-0.41	0.83	0.624	0.17	0.59	0.773
	*High*	ref.	-	-	ref.	-	-	ref.	-	-	ref.	-	-
**PM**_**10 **_**–adjusted**^**i**^	*Low*	1.20	0.66	0.068	**1.38**	**0.52**	**0.008**	0.41	1.02	0.687	0.97	0.72	0.179
	*Moderate*	**2.33**	**0.64**	**< 0.001**	**1.64**	**0.51**	**0.001**	-0.39	0.84	0.645	0.42	0.60	0.483
	*High*	ref.	-	-	ref.	-	-	ref.	-	-	ref.	-	-
**NO**_**2 **_**–adjusted**^**j**^	*Low*	1.08	0.67	0.110	**1.36**	**0.53**	**0.011**	0.66	0.93	0.481	0.38	0.66	0.566
	*Moderate*	**2.26**	**0.65**	**0.001**	**1.65**	**0.52**	**0.001**	-0.28	0.83	0.735	0.18	0.59	0.762
	*High*	ref.	-	-	ref.	-	-	ref.	-	-	ref.	-	-
**Noise annoyance-adjusted**^**k**^	*Low*	1.02	0.63	0.106	**1.06**	**0.50**	**0.033**	0.70	0.81	0.385	0.49	0.57	0.387
	*Moderate*	**2.18**	**0.63**	**0.001**	**1.44**	**0.50**	**0.004**	-0.20	0.81	0.806	0.21	0.57	0.711
	*High*	ref.	-	-	ref.	-	-	ref.	-	-	ref.	-	-

^a^. Greenness tertiles are different in Munich and Wesel areas. Munich: Low (NDVI < 0.307), Moderate (0.307 ≤ NDVI < 0.377), High (NDVI ≥ 0.377). Wesel: Low (NDVI < 0.382), Moderate (0.382 ≤ NDVI < 465), High (NDVI ≥ 0.465).

^b^. ß: Regression coefficient.

^c^. SE: Standard error.

^d^. Adjusted model is adjusted for study, sex, age, BMI, season of BP measurements, parental education and parental hypertension.

^e^. Temperature-adjusted model: Adjusted model and further adjustment for mean daily temperature on the day of BP measurements. The variable for the season of BP measurements has been removed.

^f^. Altitude-adjusted model: Adjusted model and further adjustment for altitude of residence.

^g^. Urbanisation-adjusted model: Adjusted model and further adjustment for tertiles of population density in a 5,000 m buffer around the residence.

^h^. PM_2.5_-adjusted model: Adjusted model and further adjustment for PM_2.5_ concentration at residence.

^i^. PM_10_-adjusted model: Adjusted model and further adjustment for PM_10_ concentration at residence.

^j^. NO_2_-adjusted model: Adjusted model and further adjustment for NO_2_ concentration at residence.

^k^. Noise annoyance-adjusted model: Adjusted model and further adjustment for level of noise annoyance.

Bold text indicates statistical significance at the 5 % level.

Accounting for the time of BP measurements and excluding children who had lived at their home address for less than one year did not change the estimates (data not shown). Associations stratified by moving behaviour did not yield any consistent patterns (data not shown). The estimates were robust to the inclusion of physical activity (data not shown) to the models. Furthermore, pooled and study-specific associations were similar when alternative cloud-free days for the year 2006 were used to assess NDVI values (data not shown).

## Discussion

Lower levels of residential greenness were positively associated with higher systolic and diastolic BPs in 10 year-old German children. The observed associations were robust to model adjustments for environmental stressors, such as ambient temperature and air pollution, noise annoyance, altitude and level of urbanisation. However, when stratified by study area, the associations were only significant in the urban Munich study area. Risk estimates were not significantly different from null in the rural Wesel study area.

We are the first to investigate associations between objectively assessed residential greenness and BP in children using an observational study design. The few previous studies on this topic have used experimental study designs and are limited to adults [7-12]. These studies, conducted in both laboratory and natural settings, have aimed to assess physiological stress reductions induced by walking in green spaces or simply seeing greenness. Ulrich et al. conducted the first such study in 120 undergraduate volunteers at the University of Delaware, USA [12]. In this study, the systolic BP of subjects who watched a videotape of natural environments after being exposed to a stressor decreased faster and more consistently than the systolic BP of participants who watched a videotape of urban environments. This finding was replicated in 160 college-age participants from the USA [11]. Hartig et al. [10] conducted a study among 112 young students from the University of California, USA and found that after conducting tasks, sitting in a room with views of trees induced a more rapid reduction in diastolic BP compared to sitting in a room with no view. Moreover, walking in a nature reserve promoted a greater initial BP decline and lower BP during walking compared to conducting these same tasks in urban surroundings. A recent review, which summarized the findings of the beneficial effects of “Shinrin-yoku” (taking in the atmosphere of the forest) on BP [9], reported results from twenty-four field experiments conducted across Japan among 280 young adults. This review stated that forest environments promote lower systolic and diastolic BP compared to city environments [9]. Li et al. [8] reported that a day trip to the forest park in Tokyo, Japan, reduced the BPs of 16 male participants. Finally, viewing nature scenes prior to a stressor efficiently decreased BP during the recovery period in 23 participants from Great Britain, a result which also demonstrates a potential buffering effect of greenness [7]. All aforementioned studies have reported positive effects of green spaces and greenness on BP. Together, their findings on BP and other physiological measures (heart rate, heart rate variability, cortisol levels) confirm that exposure to nature induces parasympathetic activity during the recovery from a stressor, which is consistent with the psychoevolutionary theory suggested by Ulrich [6]. Despite the lack of similar experimental studies on children, as well as any evidence from observational studies, we hypothesize that the observed association between residential greenness and BP in children in the current study is in line with this previous existing evidence and thus, with the psychoevolutionary theory. This is further supported by the fact that we also observed an association with pulse rate (PR) in the Munich study area (data not shown), although adjustment for this factor only slightly attenuated the risk estimates between greenness and BP. Despite the small effect sizes of the reported associations between greenness and BP in the current study (and in previous experimental studies), and thus the uncertain clinical impact,, our findings are nonetheless valuable as they provide further evidence of the biological mechanisms linking green vegetation exposure to health.

We are not able to explain why moderate levels of greenness appeared to increase BP more than low levels of greenness. It can be assumed that high greenness might stand for very green surroundings, low for built areas and moderate for a mixed land use. One can thus speculate that a neighbourhood without any vegetation whatsoever may not be attractive for children, which may bias the estimates. Future research is needed to determine whether this hypothesis is true.

In the analyses stratified by study area, associations between greenness and BP were only significant in the more urbanised Munich study area. This result might indicate that children living in urban regions, which generally lack vegetation, might benefit more from high residential greenness than children living in rural areas. Our findings may thus be especially relevant for policy makers and urban planners designing urban environments. Several previous studies have also reported that the degree of urbanisation may have a modifying effect on associations between green spaces and health benefits [39,40]. Our research group also recently observed differential associations between greenness and allergic outcomes in the same two study areas included in the current analysis [31].

### Limitations

Given the general lack of studies investigating the influence of greenness on BP (specifically, in children), the results of this cross-sectional study should be interpreted cautiously. Causality cannot be inferred and the observed associations may be due to chance or residual confounding, especially given the null findings observed for the Wesel study area. However, associations were similar after controlling for several environmental stressors and when greenness levels were assessed using alternative satellite images taken during cloud-free days in 2006. Due to loss of follow-up over time of the birth cohorts, there is also a potential for selection bias. Families with a lower level of education and income were less likely to participate in the 10 year follow-up. Thus, children with a lower socioeconomic status (SES) are under-represented in this analysis. Nevertheless, we cautiously adjusted our models for parental education level, which represents SES. In our study, we used the common vegetation indicator NDVI to objectively estimate residential greenness. NDVI does not allow different types of vegetation to be distinguished and is sensitive to atmospheric effects, clouds and types of soil [17]. Also, the Landsat 5 TM satellite imagery is only accurate at a resolution of 30 m, which may not allow small scale effects of greenness on BP to be captured. A further limitation is that although three repeated BP readings are usually recommended [32], only two were available for this study population. Moreover, using automated cuffs can overestimate BP in children [41]. Nevertheless, the BP measurement protocol used for this study population was the same as used in the German population-based German Health Interview and Examination Survey for Children and Adolescents (KiGGS 2003–2006) survey [23]. Finally, we were unable to control for area level socioeconomic indicators, greenness levels around schools, indoor greenness (i.e. houseplants), other physiological measurements (e.g., heart rate variability, cortisol), psychological state and fitness of children and the time the children spent outdoors in their neighbourhood, and how the children may use their neighbourhood (which activities), all of which might represent sources of residual confounding.

## Conclusions

Lower residential greenness was positively associated with higher BP in 10 year old German children. This association was independent from potential additional effects of environmental stressors. We recommend this association be further investigated in both children and adults and in different geographical areas that vary in urbanisation level. Area-level SES information and time spent in the neighbourhood should also be considered.

## Competing interests

The authors declare that they have no competing interests.

## Authors’ contributions

IM conducted the GIS work for the Munich study area, analyzed the data and wrote the paper. ET assisted with the data analyses and the interpretation of the results, and critically revised the manuscript. EF assisted with the interpretation of the results and critically revised the manuscript. DS conducted GIS work for the Wesel study area. DB, SK, AvB and CPB have made substantial contributions to the conception, design and acquisition of the data. JH has made substantial contributions to the conception, design and acquisition of the data and supervised the work. All authors read and approved the final manuscript.

## Pre-publication history

The pre-publication history for this paper can be accessed here:

http://www.biomedcentral.com/1471-2458/14/477/prepub

## Supplementary Material

Additional file 1: Figure S1 GAM-plots for the associations between residential greenness (NDVI) and blood pressure. Models adjusted for study, sex, age, BMI, season of BP measurements, parental education, parental hypertension and study centre **(A and D)**. Click here for file
