# Supplementary material for: A cross-sectional analysis of the effects of residential greenness on blood pressure in 10-year old children: results from the GINIplus and LISAplus studies
Source: BMC Public Health. 2014 May 20;14:477. doi: 10.1186/1471-2458-14-477 (PMC4035901; doi:10.1186/1471-2458-14-477)
Supplement: Additional file 1: Figure S1 — GAM-plots for the associations between residential greenness (NDVI) and blood pressure. Models adjusted for study, sex, age, BMI, season of BP measurements, parental education, parental hypertension and study centre (A and D). [file 1471-2458-14-477-S1.docx]

**
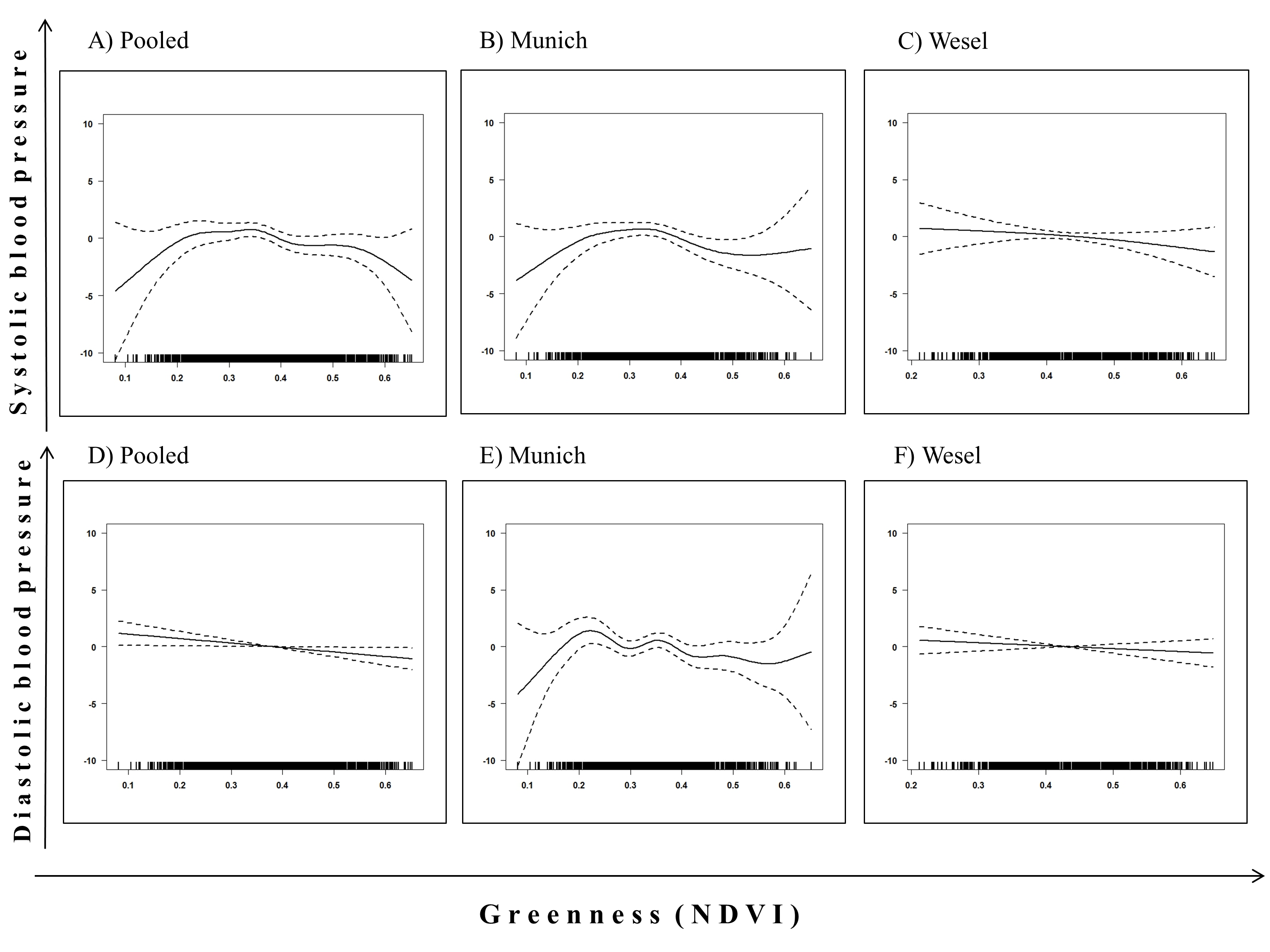
**

**Additional figure 1 GAM-plots for the associations between residential greenness (NDVI) and blood pressure.**

Models adjusted for study, sex, age, BMI, season of BP measurements, parental education, parental hypertension and study centre (A and D).
